# Supplementary material for: Predictors of Assessment of Spondyloarthritis International Society (ASAS) Health Index in Axial Spondyloarthritis and Comparison of ASAS Health Index between Ankylosing Spondylitis and Nonradiographic Axial Spondyloarthritis: Data from the Catholic Axial Spondyloarthritis COhort (CASCO)
Source: J Clin Med. 2019 Apr 5;8(4):467. doi: 10.3390/jcm8040467 (PMC6518358; doi:10.3390/jcm8040467)
Supplement: Supplementary file 1 [file jcm-08-00467-s001.pdf]

Supplement Table 1. Items constituting ASAS HI

1. Pain sometimes disrupts my normal activities.

□ I agree □ I do not agree

2. I find it hard to stand for long.

□ I agree □ I do not agree

3. I have problems running.

□ I agree □ I do not agree

4. I have problems using toilet facilities.

□ I agree □ I do not agree

5. I am often exhausted.

□ I agree □ I do not agree

6. I am less motivated to do anything that requires physical effort.

□ I agree □ I do not agree

7. I have lost interest in sex.

□ I agree □ I do not agree □ Not applicable, I do not want to answer

8. I have difficulty operating the pedals in my car.

□ I agree □ I do not agree □ Not applicable, I cannot / do not drive

9. I am finding it hard to make contact with people.

□ I agree □ I do not agree

10. I am not able to walk outdoors on flat ground.

□ I agree □ I do not agree

11. I find it hard to concentrate.

□ I agree □ I do not agree

12. I am restricted in traveling because of my mobility.

□ I agree □ I do not agree

13. I often get frustrated.

□ I agree □ I do not agree

14. I find it difficult to wash my hair.

□ I agree □ I do not agree

15. I have experienced financial changes because of my rheumatic disease.

□ I agree □ I do not agree

16. I sleep badly at night.

□ I agree □ I do not agree

17. I cannot overcome my difficulties.

□ I agree □ I do not agree
